# Dual Method Use among Postpartum HIV-Infected and HIV-Uninfected Malawian Women: A Prospective Cohort Study

**DOI:** 10.1155/2017/1475813

**Published:** 2017-07-18

**Authors:** Dawn M. Kopp, Jennifer H. Tang, Gretchen S. Stuart, William C. Miller, Michele S. O'Shea, Mina C. Hosseinipour, Phylos Bonongwe, Mwawi Mwale, Nora E. Rosenberg

**Affiliations:** ^1^UNC Project-Malawi, Tidziwe Centre, Private Bag A-104, Lilongwe, Malawi; ^2^UNC Department of Obstetrics & Gynecology, 101 Manning Drive, Chapel Hill, NC 27514, USA; ^3^UNC Department of Epidemiology, 135 Dauer Drive, Chapel Hill, NC 27599, USA; ^4^Division of Epidemiology, Ohio State University, 1841 Neil Avenue, Columbus, OH 43210, USA; ^5^UNC Department of Medicine, 125 MacNider Hall, Chapel Hill, NC 27599, USA; ^6^Department of Obstetrics & Gynaecology, Malawi College of Medicine, Private Bag 360, Chichiri, Blantyre, Malawi; ^7^Bwaila Hospital, Lilongwe District Health Office, Lilongwe, Malawi

## Abstract

Dual method use, use of condoms plus another effective contraceptive method, is important in settings with high rates of unintended pregnancy and HIV infection. We evaluated the association of HIV status with dual method use in a cohort of postpartum women. Women completed baseline surveys in the postpartum ward and telephone surveys about contraceptive use 3, 6, and 12 months later. Nonpregnant women who completed at least one follow-up survey were eligible for this secondary analysis. Prevalence ratios were calculated using generalized estimating equations. Of the 511 sexually active women who completed a follow-up survey, condom use increased from 17.6% to 27.7% and nonbarrier contraceptive use increased from 73.8% to 87.6% from 3 to 12 months after delivery. Dual method use increased from 1.0% to 18.9% at 3 to 12 months after delivery. Dual method use was negligible and comparable between HIV-infected and HIV-uninfected women at 3 months but significantly higher among HIV-infected women at 6 months (APR = 3.9, 95% CI 2.2, 7.1) and 12 months (APR = 2.7, 95% CI 1.7, 4.3). Dual method use was low but largely driven by condom use among HIV-infected women at 6 and 12 months after delivery.

## 1. Introduction

Unintended pregnancy and HIV infection are common in sub-Saharan Africa. Early initiation and continuation of effective contraception during the first 12 months after delivery can help prevent unintended pregnancy as most women do not wish to become pregnant in the first two years after childbirth [1, 2]. Use of family planning methods also decreases short interpregnancy intervals, which in some studies have been shown to increase the risk of adverse neonatal outcomes [3].

Dual method use is defined as using a barrier method, such as condoms, with another nonbarrier contraceptive method (pills, injection, implant, intrauterine contraception (IUC), or sterilization) to prevent sexually transmitted infections (STIs) and unintended pregnancy. Dual method use prevents HIV acquisition among HIV-uninfected women [4]. The risk of HIV acquisition is still high for HIV-uninfected women after their first delivery. Though 65% of women in Malawi have given birth by the time they are 20 years old, the HIV prevalence is nearly 5 times higher for Malawian women aged 30–34 as compared to 15–19 [5]. Given a total fertility rate of 5.7, it is likely that many of these infections are occurring between and during subsequent pregnancies. Dual method use also prevents transmission from HIV-infected women to their uninfected partners, while simultaneously lowering the chance of unintended pregnancy. The impact of HIV status on dual method use among postpartum women in sub-Saharan Africa is unknown.

Understanding factors associated with dual method use during the first year after delivery can help direct interventions to increase the use of effective family planning methods and prevent new HIV infections during this critical time period. Therefore, our objectives are to characterize the association between HIV status and contraceptive method mix, compare dual method uses among sexually active HIV-infected and HIV-uninfected women at three time points in the year after delivery, and compare nonbarrier contraceptive method mix between dual method users and nondual method users.

## 2. Materials and Methods

### 2.1. Study Setting and Population

This study is a secondary analysis of a prospective cohort study of postpartum Malawian women [1]. The primary study objective assessed whether HIV status is associated with fertility desire and knowledge of IUC and the contraceptive implant. A convenience sample of approximately 630 postpartum women enrolled at a 1 : 2 ratio of HIV-infected to HIV-uninfected women was determined to be necessary to evaluate this objective. The results of the primary study objective have already been published [1]. Ethical approval was obtained from the University of North Carolina School of Medicine Institutional Review Board (IRB) and the National Health Sciences Research Committee of the Malawi Ministry of Health.

Women were recruited from the postpartum unit of Bwaila Hospital, a government district hospital in Lilongwe, Malawi, with over 14,000 deliveries per year. Approximately 11% of women who deliver at Bwaila are HIV-infected. Hormonal and intrauterine contraception were not routinely offered prior to 6 weeks postpartum at this facility during the study period, but contraceptive uptake is encouraged for women attending postpartum visits 6 weeks after delivery and at other healthcare visits. In a separate study of women in Lilongwe district, approximately 57% of women utilized postnatal services within 6 weeks of delivery [6].

### 2.2. Study Design

Criteria for inclusion in the main cohort were as follows: current admission to the postpartum ward at Bwaila Hospital, age 18–45 years, live birth at greater than 28 weeks' gestation, fluency in English or Chichewa (the local language), access to a working phone number, and willingness to be contacted by phone for up to one year postpartum. Eligible women provided informed consent and completed a 30-minute baseline survey focused on demographics, reproductive health history, family planning preferences, and knowledge of IUC and implant. Women were eligible for this secondary analysis if they were recruited into the cohort and had completed at least one follow-up survey. Women who reported abstinence as their current contraceptive method were excluded from the dual method use analysis. Women with inconsistent or missing data at a given survey or who reported being pregnant were censored at that time point.

Follow-up surveys were conducted by phone at 3, 6, and 12 months after delivery and focused on contraceptive uptake and continuation. Participants also received reminder phone calls 9 months after delivery, but no survey was administered. Women who could not be contacted for a follow-up survey were generally not contacted to complete subsequent surveys, as it was assumed that the participant could no longer be reached at that phone number. However, one woman, who had not completed either the 3- or 6-month survey, was inadvertently called and completed the 12-month survey.

The two main variables of interest were baseline HIV status and dual method use. HIV testing is performed on all Malawian women during antenatal care unless they opt out. HIV status was determined by verifying the participant's health passport (a government-issued personal medical record booklet kept by the patient) with the participant's permission at time of enrollment. Contraceptive use was based on participants' self-report at each follow-up survey. Women could report more than one contraceptive method at each survey. Nonbarrier contraceptive methods available in Malawi include combined oral contraceptive pills, progestin-only contraceptive pills, the depot medroxyprogesterone acetate injectable, etonogestrel and levonorgestrel implants, the copper IUC, and both female and male surgical sterilization. Dual method use was defined as report of male or female condoms and another nonbarrier contraceptive method use at the same survey. Women were classified as using no method/breastfeeding if they did not report specifically that they were using abstinence as their contraceptive method and also did not report use of any hormonal or intrauterine contraceptive method at the same survey. Women using the contraceptive implant, IUC, or sterilization were grouped together under long-acting methods when comparing contraceptive method mix.

### 2.3. Statistical Analysis

Pearson's *χ*^2^ tests were used to compare distributions of contraceptive method mix between HIV-infected and HIV-uninfected women and between dual and nondual method users. We used generalized estimating equations with a Poisson distribution, a log link, an exchangeable correlation structure, and robust variance. These models were used to estimate predicted probabilities and 95% confidence intervals (CI) of hormonal or intrauterine contraceptive use, condom use, and dual method use. We also used these models to estimate the effect of HIV status on dual method use at the 3, 6, and 12 months after delivery. We used directed acyclic graphs to identify potential confounders for HIV status and dual method use to include in the adjusted models. All data were double-entered into a REDCap database, cleaned, merged, exported, and analyzed using Stata Version 13.0 (StataCorp, College Station, TX).

## 3. Results

### 3.1. Study Population

**Figure 1:**
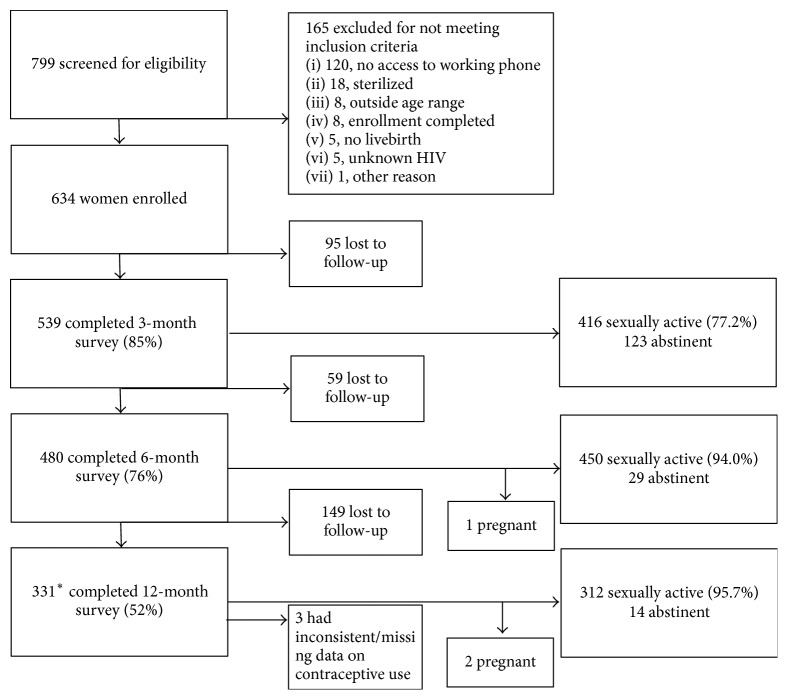
Flow diagram of survey completion and sexual activity. ^*∗*^One respondent, who had not completed the 3-month or the 6-month survey, completed the 12-month survey.

Between May 2013 and September 2013, 634 participants enrolled and completed the baseline survey (Figure 1). Follow-up data were collected from 539 (85.0%), 480 (75.7%), and 328 (51.7%) women at 3, 6, and 12 months, respectively. Survey completion rates were not different between HIV-infected and uninfected women at 3 months (85.2% versus 84.9%, *p* = 0.912) or 6 months (77.4% versus 72.4%, *p* = 0.169), but there were slightly less HIV-infected women completing the 12-month survey (45.7% versus 54.7%, *p* = 0.033).

Three women reported a pregnancy during their follow-up surveys and were censored at those time points: one was reported at the 6-month survey and two were reported at the 12-month survey. Three other women reported inconsistent data about contraceptive use from the 6- to 12-month survey and their data was censored at the 12-month survey. The demographics of women who completed any follow-up survey demonstrate that most women were less than 35 years old, married, and had completed primary school (Table 1). More HIV-infected women reported having 2 or more children, a desire to not have further children, an unintended last pregnancy, and an intention to use dual methods after delivery.

### 3.2. HIV Status and Contraceptive Method Mix

**Figure 2:**
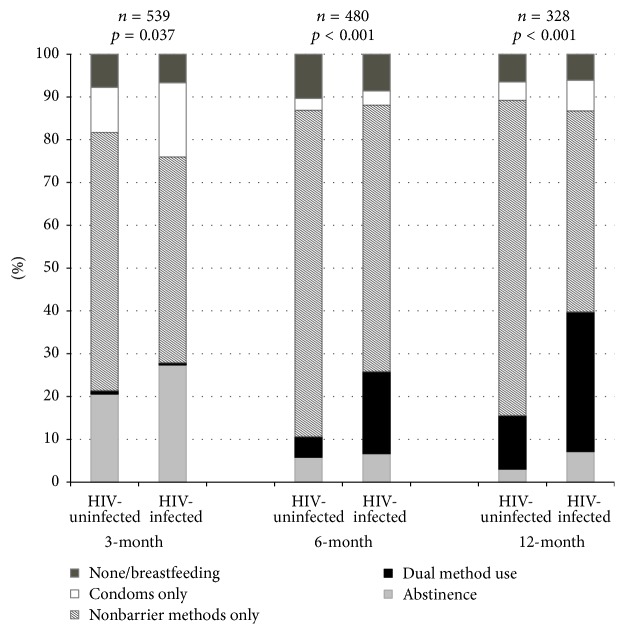
Contraceptive method use at each follow-up survey by HIV status.

The contraceptive method mix changed as time from delivery increased and differed by HIV status (Figure 2). The method mix between HIV-infected and HIV-uninfected women differed at 3 (*p* = 0.037), 6 (*p* < 0.001), and 12 months (*p* < 0.001) after delivery. Overall, HIV-infected women were more likely to use abstinence, condoms, or dual methods than HIV-uninfected women. HIV-uninfected women were more likely to use nonbarrier methods alone.

Most women resumed sexual intercourse by three months (77.2% at 3 months after delivery), but higher proportions were sexually active at 6 (94.0%) and 12 months (95.7%) after delivery. There were no differences in sexual activity between HIV-infected and uninfected women. At each survey, a majority of sexually active women reported using any form of contraception: 90.4%, at 3 months, 89.6% at 6 months, and 93.6% at 12 months. The use of nonbarrier contraceptives increased from 57.0% (307/539) at the 3-month survey to 81.2% (389/479) at the 6-month survey and to 84.3% (275/326) at the 12-month survey. Condom use (alone or with a nonbarrier contraceptive) was stable from 3 months (13.5%) to 6 months (12.3%) after delivery but increased at 12-months after delivery (23.3%). Over 90% of women reported breastfeeding at each follow-up. However, less than 10% of breastfeeding women considered this a current method of contraception for themselves and many used a separate nonbarrier method.

### 3.3. Dual Method Use

**Figure 3:**
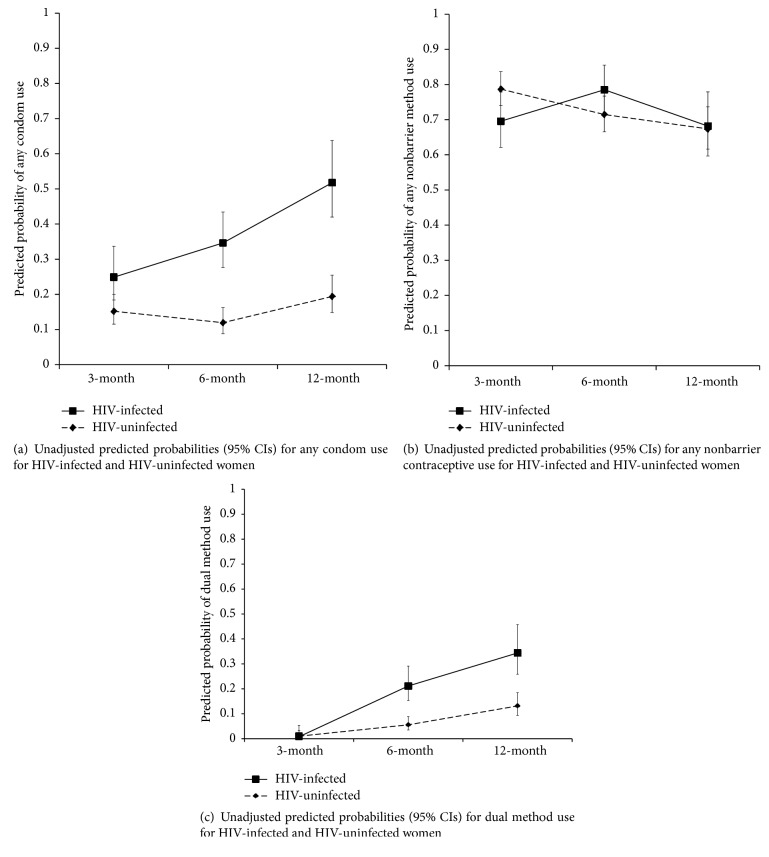
Generalized estimating equations models were used to calculate unadjusted predicted probabilities and 95% confidence intervals (CIs) at each follow-up survey for HIV-infected and HIV-uninfected women.

Comparisons of any condom use, any hormonal or intrauterine contraceptive use, and dual method use between HIV-infected and HIV-uninfected women were performed only among sexually active women at each survey (*n* = 511). Of all sexually active women, condom use increased from 17.6% to 18.0% to 27.7% and nonbarrier contraceptive use increased from 73.8% to 86.4% to 87.6% at 3, 6, and 12 months after delivery. Dual method use increased from 1.0% to 10.0% to 18.9% at 3, 6, and 12 months after delivery among sexually active respondents. HIV-infected women were more likely to use dual methods at the 6- and 12-month survey but not at the 3-month survey (Figure 3(c)). HIV-infected women were more likely to use condoms at all surveys (Figure 3(a)), but there was no difference in nonbarrier contraceptive use at any survey (Figure 3(b)). HIV-infected women were as likely as HIV-uninfected women to use dual methods at 3 months after delivery (PR = 0.8, 95% CI 0.1, 7.3) (Table 2) but were 3.8 times more likely to use dual methods at 6 months after delivery (95% CI 2.2, 6.7) and 2.6 times more likely to use dual methods at 12 months after delivery (95% CI 1.7, 4.1). This remained significant after adjustment for age, education, and parity at both 6 (aPR = 3.9, 95% CI 2.2, 7.1) and 12 months after delivery (aPR = 2.7, 95% CI 1.7, 4.3). The use of any nonbarrier methods of contraception did not meaningfully differ between HIV-infected and HIV-uninfected women at any time points, but HIV-infected women were more likely to use condoms at each time point.

### 3.4. Dual Method Use and Nonbarrier Contraceptive Mix among Users Reporting a Contraceptive Method

**Figure 4:**
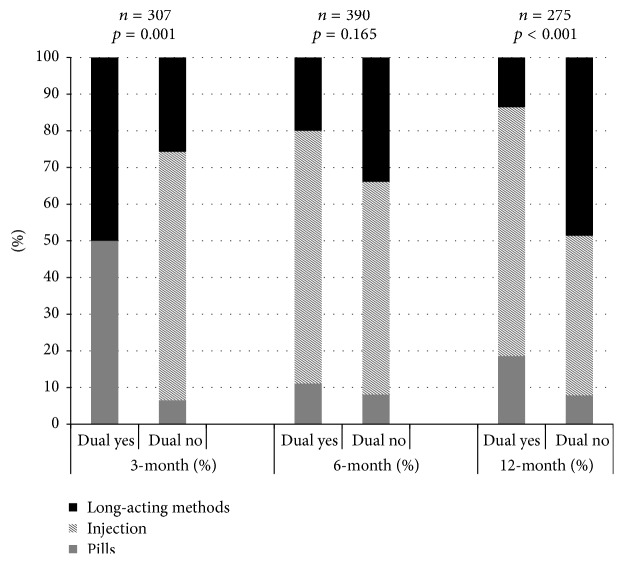
Nonbarrier contraceptive mix of dual method users and nondual method users among sexually active respondents, among users reporting a contraceptive method.

The mix of nonbarrier contraceptives differed between sexually active dual method users and nondual method users at 3 (*p* = 0.001) at 12 months after delivery (*p* < 0.001) but not at the 6 months after delivery (*p* = 0.165) (Figure 4). Among dual method users at 12 months, the most common methods were the injection (67.8%), followed by oral contraceptives (18.6%), and long-acting methods (13.6%). However, among nondual method users, long-acting methods were the most commonly reported (48.6%), followed by the injection (43.5%) and oral contraceptives (7.9%).

## 4. Discussion 

In this cohort of postpartum Malawian women, we found that while any contraceptive use in this population was high, dual method use was low but increased during the first 12 months after delivery. HIV-infected women were more likely to use dual methods than HIV-uninfected women, but this was largely driven by increased condom use. Dual method users were less likely to use long-acting methods than nondual method users at 12 months after delivery.

The prospective nature of this study is an important strength, enabling us to describe the family planning practices of women over the entire first year after delivery. Other studies involving postpartum women have only examined contraceptive use at one time point [7, 8]. However, in the course of the study, we experienced a high rate of loss to follow-up, which is a potential source of bias. Those retained may have been more motivated to use family planning methods than those who were lost as they continued to have working telephone numbers and answer study phone calls, which may be associated with a higher socioeconomic status. Additionally, less HIV-infected women completed the 12-month survey, which may have increased the proportion of HIV-infected dual method users, if contraceptive users are more likely to complete surveys. The demographics of our postpartum African cohort demonstrate women with less education, a higher rate of marriage, and similar rate of unintended last pregnancy to cohorts in South Africa [7] but higher education and a similar rate to women in a postpartum cohort of women in Nigeria [9].

Though most of our participants were sexually active by 3 months after delivery, many initially used either nonbarrier methods or condoms alone, with dual method use increasing as time from delivery increased. The low initial dual method use may be due to low perceived STI or pregnancy risk due to breastfeeding, infrequent sex, or other factors [9]. Other reasons for low utilization of dual methods during this period may be difficulty accessing other hormonal or intrauterine contraceptives during the first 6 months due to distance to a health facility, lack of method choice at the health facility, provider bias to administer less work-intensive methods to women they perceived to be at low pregnancy risk [10], or other factors. These access related factors were observed among postpartum women in Morocco, where contraceptive use was higher among women who lived closer to health centers and who had three or more contraceptive methods offered at their nearest health center [11].

The proportion of women using family planning is higher than in other published studies in Malawi. Demographic and Health Surveys (DHS) data from Malawi shows that approximately 14% of women are using modern methods of contraception by 3 months after delivery; however this was higher among urban women [12]. A recent study in rural, northern Malawi reported that 28.4% of women used modern methods by 6 months and 45.8% used modern methods by 12 months after delivery [13]. However, these proportions included women who reported abstinence. Among sexually active, menstruating women, 78% were using modern methods of contraception at 6–9 months after delivery, similar to our results.

The relationship between HIV status and contraceptive use has been observed in other studies of postpartum women. While dual method use was low (1–19%) among the women in our study, it is comparable to the range (0.25–15%) [14–16] reported in other postpartum African populations. In a cross-sectional study of postpartum South African women, HIV-infected women more frequently report condom use [12] than HIV-uninfected women. In other settings, HIV-infected women reported higher usage of any contraceptive method when compared to HIV-uninfected women [17, 18]. In our population, we found that condom use and dual method use were associated with HIV status but not nonbarrier contraceptive use. Additionally, condom and dual method use may be influenced by other factors for which data were not collected, such as HIV status of the partner, women who may have been female sex workers during the study period, healthcare provider bias toward certain contraceptive methods, relationship security, intimate partner violence, partner disclosure/communication, or ART use of the woman and partner [19, 20].

The outcome of contraceptive use was by self-report and could not be confirmed with healthcare files due to the design of the study. The association with reported condom use and HIV status may be due to social desirability/reporting bias leading women to overreport condom use. If this bias was more prevalent among HIV-infected women, it may have falsely increased the dual method use data for these women and strengthened the association of HIV status and dual method use. A way to examine this is with biomarker verification with spermatozoa or prostate specific antigen, markers of semen exposure. Using these methods, overreporting of condom use has been seen in HIV-infected American women [21] but not in a study of discordant couples in Kenya where most women were the HIV-infected partner [22]. Use of hormonal contraception has not been shown to be associated with overreporting of condom use when users are compared to nonusers of hormonal contraception [21, 23]. Even if self-report is an imperfect measure of condom use, it has been strongly associated with lower HIV acquisition throughout sub-Saharan Africa [24, 25].

In this analysis, we are able to compare the outcomes by baseline HIV status. However, HIV status is not static and high HIV incidence has been described in the postpartum period [26]. Women or their partners in this cohort may have become HIV-infected over the follow-up period, which could have increased their condom use and lessened the association of baseline HIV status and dual method use. Due to our study design, we were unable to assess incident HIV infection in this cohort. Additionally, among HIV-infected women, time from HIV diagnosis was not accounted for, which has been shown to influence condom use [27]. Additionally, we only evaluated contraceptive use at three time points and therefore were unable to account for inconsistent use or gaps in contraceptive coverage. Other studies have used contraceptive calendars [28, 29] to collect month-by-month data on contraceptive use or asked participants to report condom and contraceptive use for each sexual act [30].

Further studies should focus on studying barriers to dual method use and behavioral interventions to increase dual method use in a postpartum population. The optimal nature and timing of these interventions have not been established. A recent Cochrane review on interventions to increase dual method use did not include any studies involving postpartum interventions but did find that some educational programs showed a decrease in the incidence of STIs [31]. More research is needed to understand optimal ways to promote dual method use and increase uptake in postpartum women.

## 5. Conclusions

The use of dual methods in women after delivery was associated with HIV status but low overall, putting postpartum women at risk of STIs and pregnancy. The low dual method use in this at-risk population identifies an opportunity for public health researchers, providers, and policymakers to design and evaluate interventions to increase this use. Further understanding and addressing the barriers to dual method use, especially among HIV-uninfected women, are important to the design and implementation of effective comprehensive pregnancy and HIV prevention programs.

## Figures and Tables

**Table 1:** Characteristics of women responding to follow-up surveys by HIV status (*n* = 539).

	HIV-infected (*n* = 179)	HIV-uninfected (*n* = 360)
Age (years) *n* (%)		
18–24	59 (33.0)	189 (52.5)
25–34	95 (53.1)	151 (41.9)
≥35	25 (14.0)	20 (5.6)
Relationship status *n* (%)		
Married	167 (93.3)	342 (95.0)
Unmarried	12 (6.7)	18 (5.0)
Education *n* (%)		
None or some primary	59 (33.0)	82 (22.8)
Primary/some secondary	78 (43.6)	165 (45.8)
Secondary and beyond	42 (23.5)	113 (31.4)
Trouble with food, clothing, or medications *n* (%)		
Yes	106 (59.2)	186 (51.7)
No	72 (40.2)	173 (48.1)
Missing	1 (0.6)	1 (0.3)
Living children *n* (%)		
1	44 (24.6)	158 (43.9)
2-3	93 (52.0)	151 (41.9)
≥4	42 (23.5)	51 (14.2)
Desiring any more children *n* (%)		
Yes	73 (40.8)	261 (72.5)
No	101 (56.4)	91 (25.3)
Do not know	4 (2.2)	6 (1.6)
Missing	1 (0.6)	2 (0.6)
Most recent pregnancy intention *n* (%)		
Intended	91 (50.8)	231 (64.2)
Unintended/do not know	88 (49.2)	129 (35.8)
Intending to use dual method		
Yes	95 (53.1)	124 (34.4)
No	84 (46.9)	236 (65.6)

**Table 2:** Prevalence ratios (PRs) of dual method, nonbarrier contraceptive, and condom use by HIV status at each follow-up survey among sexually active respondents.

	3-month survey		6-month survey		12-month survey	
	Unadjusted PR (95% CI)	Adjusted PR (95% CI)^1^	Unadjusted PR (95% CI)	Adjusted PR (95% CI)^1^	Unadjusted PR (95% CI)	Adjusted PR (95% CI)^1^
*Any condom use*						
HIV-uninfected	1	1	1	1	1	1
HIV-infected	**1.6 (1.1, 2.5)**	**1.7 (1.1, 2.5)**	**2.9 (2.0, 4.2)**	**2.9 (2.0, 4.3)**	**2.7 (1.9, 3.8)**	**2.7 (1.9, 3.8)**
*Any nonbarrier contraceptive use*						
HIV-uninfected	1	1	1	1	1	1
HIV-infected	0.9 (0.8, 1.0)	0.9 (0.8, 1.1)	1.1 (1.0, 1.2)	1.10 (1.0, 1.2)	1.0 (0.9, 1.2)	1.0 (0.9, 1.2)
*Dual method use*						
HIV-uninfected	1	1	1	1	1	1
HIV-infected	0.8 (0.1, 7.3)	0.8 (0.1, 7.5)	**3.8 (2.2, 6.7)**	**3.9 (2.2, 7.1)**	**2.6 (1.7, 4.1)**	**2.7 (1.7, 4.3)**

^1^Adjusted for age, education achieved, and parity.
